# Vitamin D Deficiency—A Clinical Spectrum: Is There a Symptomatic Nonosteomalacic State?

**DOI:** 10.1155/2010/521457

**Published:** 2009-09-15

**Authors:** Amar Kanekar, Manoj Sharma, V. R. Joshi

**Affiliations:** ^1^Health Promotion & Education, University of Cincinnati, P. O. Box 210068, Cincinnati, OH 45221-0068, USA; ^2^Division of Rheumatology, P.D. Hinduja Hospital and Medical Research Centre, Veer Savarkar Marg, Mahim, Mumbai 400016, India

## Abstract

Vitamin D deficiency is not uncommon even in the sunny land of India. Lack of sun exposure and inadequate oral intake are both responsible for vitamin D deficiency. This article provides a retrospective, examining the effects of Vitamin D deficiency in 71 patients. The study's inclusion criterion was low vitamin D level combined with musculoskeletal symptoms but without the presence of osteomalacia. All patients in this study were suspected to have vitamin D deficiency. The data were retrieved from the case-charts of patients seen between 1996 and 2001 at the rheumatology services of Hinduja Hospital, Mumbai, India. This study found no correlation between Vitamin D levels and symptoms, or between the severity of Vitamin D deficiency and the number of symptoms displayed. Subclinical vitamin D deficiency or preosteomalacic state was the term coined for individuals with vitamin D deficiency producing nonspecific musculoskeletal symptoms in the absence of clinical osteomalacia.

## 1. Introduction

Vitamin D costs nothing. It is freely available to any one who allows adequate exposure of skin to the ultraviolet radiation present in the sunrays. However, vitamin D deficiency is not uncommon even in the sunny land of India. The clinical syndrome vitamin D deficiency consists of osteomalacia in adults and rickets in children. Other manifestations of vitamin D deficiency include nonspecific backache, joint pains, and generalized body ache. With the availability of a method to accurately measure 25-hydroxyvitamin D [25(OH) D], a state of hypovitaminosis D without overt signs and symptoms of vitamin D deficiency has become apparent [1, 2]. The amount of vitamin D provided by sun exposure and diet is difficult to determine as the duration and intensity of sunlight exposure depends on age, clothing, and sunscreens [3].

The insufficient intake of vitamin D has been implicated as one of the factors in the development of bone disease [4–6]. Various elements contribute to this insufficient vitamin D intake such as skin pigmentation [7], which presumably interferes with ultraviolet ray transmission through epidermis, genetic factors [8, 9], social customs such as avoidance of sun exposure, consumption of chapattis (flattened rounded wheat bread) that is high in phytates which bind calcium in the gut and interfere with calcium absorption [10], lack of sun exposure and malnutrition to name a few important contributing factors.

Lack of exposure to the sun is a leading cause of vitamin D deficiency. This is exemplified by the high prevalence of vitamin D deficiency in house bound and elderly patients [11–13].

Another important factor is malabsorption of Vitamin D [14, 15].

While osteomalacia and rickets have been studied extensively, there is little information on vitamin D deficiency without obvious bone disease.

The aims and objectives of this study were (a) to study the clinical symptoms and signs of vitamin D deficiency and their correlation with 25-hydroxyvitamin D [25(OH) D] levels, (b) to study the bone profile of patients of hypovitaminosis D, and (c) to observe the response to treatment with vitamin D on these individuals.

## 2. Materials and Methods

This study involves a retrospective chart analysis of 71 patients. The inclusion criterion for the study was low vitamin D level without osteomalacia. The data are derived from case charts, obtained from the medical records department. The patients were seen between 1996 and 2001 at the rheumatology services of the Hinduja Hospital and Medical Research Centre, Mumbai, India. Mumbai is a city which lies at a latitude 18°55′ N in the western part of India.

A proforma was prepared to record relevant details (Appendix A-proforma: profile of vitamin D deficiency). This included details regarding symptom and signs, dietary history, duration and severity of illness, associated illnesses, sun exposure, renal disease, gastrointestinal tract disease and intake of anticonvulsants such as diphenylhydantoin sodium. The treatments given to these patients were recorded and their responses to treatment were noted. For the details not available in the medical chart, an attempt was made to contact the patients on telephone or in person to get complete information. A questionnaire was also posted to all the patients included in the study. The data so acquired were analyzed.

## 3. Results

The demographics of the 71 patients under study showed that 87% were females. The age-range was wide (21–80 years). Of these 38% (*n* = 27) were in the age group of 31–40 years. Table 1 reflects these demographics.

The frequency of various “rheumatic” symptoms is shown in Table 2. Arthralgia and backache were present in over 50% of these cases. Vitamin D deficiency in this population, while symptomatic, was without any of florid signs (63% showed no tenderness—as shown in Table 3).

The bone profile of patients with vitamin D deficiency is shown in Table 4. The number of diseases associated with hypovitaminosis D is shown in Table 5. The radiological data showing various vitamin D associated changes in the study's patients are shown in Table 6.

Taking the normal range of vitamin D, 25(OH) D in our laboratory as 9.9–41.5 ng/mL, we classified our patients into 3 grades of vitamin D deficiency. The grades were mild 25-hydroxyvitamin D [25(OH) D] levels—6 to 9.9 ng/mL, moderate 25-hydroxyvitamin D [25(OH) D] levels—2 to 5.9 ng/mL, and severe 25-hydroxyvitamin D [25(OH) D] levels < 2 ng/mL. The distribution of severity of vitamin D amongst these patients is shown in Table 7 using these parameters. Vitamin D deficiency is variably defined as a 25(OH) D level less than 10–15 *μ*g/L [16, 17].

## 4. Discussion

The commonness of vitamin D deficiency in an otherwise healthy population was an eye-opener as well as a teaser. At the Hinduja Hospital and Medical Research Center, Mumbai, India, the awareness of vitamin D deficiency started with appreciation of vitamin D deficiency as a complicating factor in rheumatic diseases. A case is briefly described to make the point [18].

A 59year-old male patient with ankylosing spondylitis of more than 30 years duration was admitted with a recurrence of left-sided hemiparesis and a recent increase in “joint pains”. He had diabetes for the past 10 years which was controlled on oral hypoglycemic agents. A year before his admission, he had developed left-sided hemiparesis from which he had recovered completely. The increase in joint pains started six months before he was admitted but he reported no swelling or stiffness at that time.

Clinical examination revealed stooped posture and a stiff spine. He had a severe limitation of cervical, thoracic, and lumbar spine movements. There was no evidence of synovitis of peripheral joints, but he had severe diffuse bony tenderness, especially of his spine and ribs. Investigations revealed the following statistics: Hemoglobin: 12.2 gm/dL, White blood cell count: 16,700/mm^3^, Platelets: 2.3 lac/mm^3^, ESR: 28 mm/h, Serum calcium: 8.3 mg/dL (normal range: 8 to 10.4 mg/dL), Phosphorus 2.9 mg/dL (normal range: 2.5–4.6 mg/dL), Alkaline phosphatase: 590 iu/L (normal range: 40–120 iu/L), 25(OH) vitamin D: 4.3 ng/mL (normal range: 9.9–41.5 ng/mL) and Parathormone 333 pg/mL (normal range: 12–72 pg/mL). An X-ray of his chest revealed multiple rib fractures. A routine urine test showed trace proteins, 24 hours urinary proteins: 792 mg (0–165 mg) and creatinine 1 mg/dL (normal range: 0.6 to 1.01 mg/dL).

He was diagnosed with vitamin D deficiency-related osteomalacia with secondary hyperparathyroidism being made and he was initiated on a vitamin D treatment regimen (alpha D3 and vitamin D 60 000 units), along with one gram supplemental calcium. Within one week his pains started to decrease and were significantly more manageable in three weeks time and he was discharged. At three months followup he was painfree but declined repeat blood tests.

This man's medical case was followed by the publication of a small series of letters to the editor in the *Journal of Association of Physicians of India*. Soon patients with a variety of nonspecific musculoskeletal complaints were tested for vitamin D status. To our surprise the prevalence of vitamin D deficiency was high. Thus of the samples tested for vitamin D levels in our hospital laboratory, 80% had low serum 25-hydroxyvitamin D [25(OH) D] levels, that is, <9.9 ng/mL. It therefore became imperative that we study the significance of low vitamin D levels in adults for clinical, therapeutic, and other aspects. It is with this view that this study was undertaken. Thus the case selection was based upon the presence of low vitamin D levels in individuals attending the clinic, for various nonspecific musculoskeletal symptoms. (For comparison data from frankly osteomalacic individuals have been included. This study does not include pediatric age group. Such data were not provided.)

The present study has its inherent limitations but was thought necessary to understand the phenomenon of low vitamin D levels. All the study patients had musculoskeletal complaints and were suspected to suffer from rheumatoid arthritis, ankylosing spondylitis, or vertebral disc disease. Although it is not possible to judge the exact proportion of such patients, vitamin D deficiency is an important differential diagnosis of patients with such complaints. In rheumatology parlance, fibromyalgia and hypothyroidism closely resemble this symptomatology, and hence myalgia and nonspecific pain may be due to vitamin D deficiency [19].

Body pain and arthralgia was sometimes severe enough to limit day to day activities. Muscle weakness led to inability to get up from squatting position and waddling gait in some patients. Vitamin D deficiency is an important consideration in a patient with proximal muscle weakness, (and may additionally complicate a case of polymyositis-dermatomyositis). Vitamin D deficiency in this population was without florid signs (63% showed no tenderness). This is important, as without bony tenderness (which would suggest osteomalacia) and without proper awareness on the part of physicians, vitamin D deficiency can easily be overlooked.

### 4.1. Correlation of Vitamin D Deficiency with Symptoms

When we tried to correlate the vitamin D levels with the symptoms (as shown in Table 7), we came to the conclusion that although patients with low vitamin D levels present many nonspecific symptoms like backache, body ache, leg pain, and thigh pain, not all are osteomalacic. How does one explain the absence of clinical osteomalacia in individuals with very low vitamin D levels? Is it likely that even at low vitamin D levels, there is enough 1, 25 (OH)_2_ D_3 _ to maintain homeostasis? Although this current study does not possess the funding to answer this question, it remains an interesting line of inquiry.

There may be (over) active conversion of 25 (OH) D to 1, 25 (OH)_ 2_ D_3 _ to maintain bone mass. A parallel can be drawn with iron deficiency anemia; until body iron stores are exhausted iron deficiency does not manifest as anemia.

An attempt was made to see if the severity of vitamin D deficiency correlated with the number of symptoms recorded (as shown in Table 7). (Unfortunately, the severity of individual symptoms cannot be analyzed as this was not recorded using a visual analogue scale.) Still there does not seem to be any such correlation given that only one patient had more than four out of the seven symptoms analyzed.

We compared the records of 12 patients who were diagnosed as osteomalacic and found that 50% of the patients had vitamin D levels that measured at levels below 5 ng/mL.

### 4.2. Concept of “Subclinical” Vitamin D Deficiency

It is apparent that individuals with vitamin D deficiency may not suffer from clinical osteomalacia but can have nonspecific musculoskeletal symptoms accompanied with morbidity. Such patients could be diagnosed with “*Subclinical Vitamin D deficiency or Preosteomalacic State*”. It is likely that some may go on to develop frank nutritional osteomalacia unless treated. This is because, compared with the commonness of vitamin D deficiency, clinical osteomalacia is not a common disorder. It is difficult to guess the prevalence of vitamin D deficiency in the community, but it does seem logical to assume that osteomalacia (as well as rickets) are only the tip of the iceberg. Therefore, it is important to recognize “*Subclinical Vitamin D deficiency*”.

### 4.3. Aetiology of Vitamin D Deficiency

The common etiological factors of vitamin D deficiency are lack of exposure to sun rays (solar ultraviolet-B exposure to as much of body surface as possible produces vitamin D) and decreased intake of foods rich in vitamin D. A specific history was not recorded in all patients studied, but a majority of these patients were vegetarians, with poor intake of milk or milk products and little outdoor activity. None of the patients belonged to a low socioeconomic group and hence likely had access to plenty of food. Thus paradoxically, vitamin D deficiency may be a disease of the rich! The following case of osteomalacia illustrates this point.

The case involves a 50-year-old gentleman, a businessman and an epileptic on diphenylhydantoin sodium living in a house with drawn curtains, and traveling in a car with tainted glass. He was rarely exposed to sun rays.

In addition to this, he was a vegetarian. When seen by the physician, he was nearly crippled by back pain with frank evidence of osteomalacia. The pain was treated as spondylosis, and he was even suspected to be suffering from tuberculosis!. Vitamin D, calcium, and regular sun exposure brought him back to normalcy.

### 4.4. Response to Treatment

When we checked the response to vitamin D and calcium therapy, we found that out of 20 patients placed on calcium and/or vitamin D, 13 patients showed significant clinical improvement. We could not repeat the biochemical parameters. Also followup information was not provided for 51 of these patients, and so it is impossible to speculate about their response to treatment.

It should be noted that the cause for nonresponse to treatment in some patients may be due to factors other than vitamin D deficiency. Additionally, the symptoms these patients report, symptoms that indicate a lack of vitamin D, may also be caused by other incidental factors. For example a recent controlled study found that diffuse musculoskeletal pain may not be associated with low vitamin D and that this pain does not respond to treatment with vitamin D [19].

### 4.5. Vitamin D Deficiency Complicating Rheumatic Disorder

As already stated vitamin D deficiency can complicate a rheumatic disease and make diagnosis difficult [18, 20–23]. The following case study illustrates this point. Mrs. G, a 46-year-old female patient suffering from rheumatoid arthritis for more than 15 years had tried a few disease modifying agents at the initial stages of the disease without beneficial effects. Eight years prior to being seen, she had undergone a left hip replacement. For five of those years, she was bed ridden with deformities and complained of severe, debilitating, unbearable aches and pains. She could not even turn in bed, move her legs, or lift her arms due to the pain. She was told that her arthritis was very advanced and nothing could be done. Examination revealed that her synovitis was mild and the pains were “bony”. Vitamin D deficiency was suspected.

Investigations revealed undetectable levels of vitamin D, low serum calcium, phosphorus, and increased alkaline phosphatase.

X-rays revealed severe osteopenia accompanied by a deformed pelvis. There were no definite “*Looser's zones*.” A diagnosis of concomitant osteomalacia was made, and she was placed on three weekly bolus of 60 000 units of oral vitamin D followed by a daily supplement of 1.5 grams of calcium. Over a period of 1 month, her bone pains reduced significantly. She was able to lift her arms, eat with her own hands, and sit without support. The case was an eye-opener. In every case with pains out of proportion to clinical findings, it would be wise to check for vitamin D deficiency symptoms.

### 4.6. Limitations

Since this was a retrospective study where the study participants had to recall their dietary history, type of indoor and outdoor activities performed, and history of sun exposure, a recall bias is evident. Furthermore the data on assessment of muscle weakness were incomplete and were not evaluated by another independent rater. This was hence not reported in this study.

## 5. Conclusion

This small retrospective study shows that vitamin D deficiency is not uncommon but frank osteomalacia is uncommon. A “*Subclinical Vitamin D State*” exists which is characterized by nonspecific musculoskeletal symptoms. This state may complicate a rheumatic disorder, and it is important to consider this diagnosis as the results of treatment are most gratifying. Three conclusions can be drawn from this study: (a) vitamin D deficiency runs across the board; importantly it affects individuals in the prime of their life and affects the quality of life and the productivity of those suffering from it; (b) the preponderance of female patients compared to male patients suffering from vitamin D deficiencies possibly reflects social factors like the avoidance of milk and milk products and staying indoors; (c) a large chunk of vitamin D deficiency is “*subclinical*.” Nonspecific musculoskeletal symptoms are the only manifestations. These symptoms may masquerade as exacerbation of an existing rheumatic disorder. If estimation of vitamin D is not possible, a trail of oral vitamin D supplements and calcium is indicated to be beneficial.

There is a new and emerging body of literature showing relationship between vitamin D deficiencies and respiratory infections [24], along with obstetrical conditions such as pre-eclampsia [25]. These relationships can be explored in future studies which look at vitamin D deficiencies.

## Figures and Tables

**Table 1:** Age: wise distribution of cases (*n* = 71).

Age groups	Number of patients	Percentage
<20 years	0	0%
21–30 years	1	1%
31–40 years	27	38%
41–50 years	23	32%
51–60 years	7	10%
61–70 years	10	14%
71–80 years	3	4%
>81 years	0	0%

**Table 2:** Presenting symptoms in cases (*n* = 71).

Symptoms*	Number	Percentage
(1) Arthralgia or limitation of movements	49	69%
(2) Back Pain	36	51%
(3) Lower limb pain	18	25%
(4) Generalized body ache	14	20%
(5) Hip or thigh pain	8	11%
(6) Muscular weakness	6	8%
(7) Sciatica like symptoms	3	4%

*Some patients had more than one symptom.

**Table 3:** Presenting signs in cases (*n* = 71).

Presenting signs in cases	Number of patients
(1) No tenderness	45
(2) Tenderness at any other site	16
(3) Rib tenderness	8
(4) Pelvic tenderness	2
(5) Sternal tenderness	0

**Table 4:** Bone profile of patients with Vitamin D deficiency.

Serum levels	<Normal	Normal	>Normal	Normal range
(1) Vit D levels	71	0	0	9.9–41.5 ng/mL
(2) Serum calcium	10	61	0	8.0–10.4 mg/dL
(3) Alkaline phosphatase	2	48	21	40–120 IU/mL
(4) Phosphorus	4	66	1	2.0–5.0 mg/dL

**Table 5:** Diseases associated with hypovitaminosis D.

Associated diseases	Number of patients
(1) Renal diseases	2
(2) Seizure disorders	1
(3) Malabsorption	1
(4) GI Disease other than malabsorption	0
(5) Other diseases**	23

**Elaborated other diseases	
* *(a) Hypertension	7
* *(b) Diabetes	5
* *(c) Rheumatoid Arthritis	2
* *(d) Spondyloarthropathy	2
* *(e) Asthma	3
* *(f) Hypothyroidism	3
* *(g) Anemia	1

**Table 6:** Vitamin D associated changes in the study patients (*n* = 71).

Radiological data	Number of patients
(1) Normal	50
(2) Spondylolisthesis + Degenerative spine	9
(3) Osteopenia	7
(4) Scoliosis	1
(5) Looser's zones	1
(6) Pelvic fractures	1
(7) Sacroilitis	1
(8) Avascular necrosis	1

**Table 7:** Correlation of Vitamin D levels with symptoms. Sy: symptoms.

Vitamin D levels	7 sy.	6 sy.	5 sy.	4 sy.	3 sy.	2 sy.	1 sy.
(1) 8–9.9 ng/mL (25 patients)	0	0	0	3	3	12	7
(2) 6–7.9 ng/mL (10 patients)	0	0	0	0	2	3	5
(3) 4–5.9 ng/mL (18 patients)	0	0	0	1	3	7	7
(4) 2–3.9 ng/mL (11 patients)	0	0	1	0	2	4	4
(5) <2 ng/mL (7 patients)	0	0	0	2	2	3	0
